# Impact of surgical margins on recurrence and survival rate in patients with oral squamous cell carcinoma: A systematic review and meta-analysis

**DOI:** 10.4317/medoral.27784

**Published:** 2025-11-22

**Authors:** Andrea Rubert, Leticia Bagan, Alex Proaño, Gracia Sarrion, Jose Bagan

**Affiliations:** 1 Oral Medicine Unit, Department of Stomatology, University of Valencia, Valencia, Spain; 2 Precancer and Oral Cancer Research Group of Valencia University, Valencia, Spain

## Abstract

**Background:**

Oral squamous cell carcinoma (OSCC) accounts for approximately 90% of malignant neoplasms of the oral cavity. At early stages, the treatment of choice is surgical resection with clear margins, commonly defined as 5mm of tumor-free tissue. However, the optimal surgical margin in relation to recurrence and survival remains controversial. The objective of this study was to evaluate the impact of surgical margin status on local recurrence and overall survival in patients with OSCC through a meta-analysis.

**Material and Methods:**

An electronic search was conducted in Medline-PubMed, Web of Science, and Scopus up to January 2025. Two investigators independently selected the studies according to the inclusion criteria. The study included prospective and retrospective studies assessing patients with oral squamous cell carcinoma who underwent surgical treatment and reported data regarding surgical margin status, recurrence rates, and survival outcomes. The Newcastle-Ottawa Scale was used for non-randomized observational studies. Odds ratios were estimated with 95% confidence intervals, and forest plots were generated using random-effects or fixed-effects meta-analyses depending on heterogeneity. Sensitivity analyses and publication bias analyses were performed using funnel plots and Egger's test. All statistical analyses were conducted using Comprehensive Meta-Analysis software, version 3.0.

**Results:**

Positive margins (&lt;5mm) were significantly associated with a higher rate of local recurrence (OR=2.72; 95% CI: 2.04-3.62; p&lt;0.001), while negative margins (5mm) were linked to a 1.58 -fold increase in the probability of 5-year survival (RR=0.63; 95% CI: 0.55-0.74; p&lt;0.001).

**Conclusions:**

Surgical margin status is a prognostic factor for locoregional control and overall survival in OSCC. A cutoff value of 5mm is proposed as the optimal surgical margin.

## Introduction

Oral squamous cell carcinoma (OSCC) is the most prevalent malignant tumor of the head and neck, accounting for 90% of oral cavity cancers and representing the sixth most common malignant neoplasm worldwide (1). The treatment approach is multidisciplinary, with surgery and/or radiotherapy as the primary modalities. The selection of treatment is determined by factors including tumor size, depth, location, as well as cervical lymph node involvement and distant metastases (2).

In the early stages, surgery is considered the gold standard, aiming to excise the tumor with adequate safety margins (3), which entails removal of clinically uninvolved surrounding tissue (4). In advanced cases, chemotherapy or immunotherapy, often combined with radiotherapy, is recommended (3 , 5 , 6).

The status of surgical resection margins is considered an important predictor of tumor recurrence and patient prognosis. However, there is some controversy regarding the optimal safety margin needed to improve survival outcomes in patients with OSCC (7).

The classification of surgical margins as "clear" (&gt;5mm), "close" (1-5mm), and "involved" (&lt;1mm) was introduced in 1998 by the Royal College of Pathologists in the United Kingdom, which published guidelines for head and neck carcinomas (7). Currently, new consistent evidence is needed to clarify the classification, as some studies consider a 4mm margin optimal (7), while others define 5mm as the threshold, still classifying 5mm as "close" (8). Similarly, definitions of "involved margins" vary, with some authors considering 0mm (9), others 0-1mm (10), or &lt;2mm (11) as "involved".

Most studies agree that involved surgical margins negatively impact survival in OSCC patients (10 - 12), and recurrence rates are higher when the tumor-free margin is less than 5mm compared to margins greater than 5mm (2 , 9 , 10).

Reported recurrence rates vary widely, ranging from 9.1% (13) to 50% (10), although accurately determining the true extent of the tumor and the healthy surrounding tissue can be difficult (14).

The high recurrence rates result in significant morbidity, which, combined with the low survival rates of OSCC, represent a major global health concern (15).

The objective of this study was to evaluate the impact of surgical margins on recurrence and survival rates in patients with oral squamous cell carcinoma through a meta-analysis.

## Material and Methods

Design and Search Strategy

This systematic review was conducted in accordance with the PRISMA guidelines (16), and its protocol was registered in the International Prospective Register of Systematic Reviews (PROSPERO) (CRD420251114327).

Our systematic review was performed to answer the following PICO question:

"Are there differences in recurrence and survival rates among patients with oral squamous cell carcinoma undergoing curative surgical treatment depending on the status of surgical safety margins?"

The PICO elements were:

P (Population): Patients diagnosed with oral squamous cell carcinoma based on clinical and histopathological criteria.

I (Intervention): Curative surgical resection.

C (Comparison): Status of surgical margins.

O (Outcomes):

- O1: Recurrence of oral squamous cell carcinoma.

- O2: Patient survival rate.

A comprehensive systematic search was conducted in the Medline-PubMed, Web of Science, and Scopus databases up to January 2025, using the following keywords and MeSH terms: "Oral squamous cell carcinoma", "Squamous cell carcinoma of head and neck", "Oral cancer", "Surgical procedure", "Margins of excision", "Positive margins", "Negative margins", "Tumor free margins", "Survival", "Recurrence", and "Neoplasm recurrence".

Keywords were combined using the Boolean operators AND and OR, as well as controlled vocabulary terms (MeSH in PubMed), to obtain the most comprehensive and relevant search results.

The PubMed search strategy was: (("squamous cell carcinoma of head and neck"[MeSH Terms] OR "squamous cell carcinoma of head and neck"[MeSH Terms] OR ("mouth neoplasms"[MeSH Terms] OR ("mouth"[All Fields] AND "neoplasms"[All Fields]) OR "mouth neoplasms"[All Fields] OR ("oral"[All Fields] AND "cancer"[All Fields]) OR "oral cancer"[All Fields])) AND ("surgical procedures, operative"[MeSH Terms] OR "margins of excision"[MeSH Terms]) AND ("margins of excision"[MeSH Terms] OR (("negative"[All Fields] OR "negatively"[All Fields] OR "negatives"[All Fields] OR "negativities"[All Fields] OR "negativity"[All Fields]) AND ("margins of excision"[MeSH Terms] OR ("margins"[All Fields] AND "excision"[All Fields]) OR "margins of excision"[All Fields]))) AND ("neoplasm recurrence, local"[MeSH Terms] OR ("recurrance"[All Fields] OR "recurrence"[MeSH Terms] OR "recurrence"[All Fields] OR "recurrences"[All Fields] OR "recurrencies"[All Fields] OR "recurrency"[All Fields] OR "recurrent"[All Fields] OR "recurrently"[All Fields] OR "recurrents"[All Fields]) OR ("mortality"[MeSH Subheading] OR "mortality"[All Fields] OR "survival"[All Fields] OR "survival"[MeSH Terms] OR "survivability"[All Fields] OR "survivable"[All Fields] OR "survivals"[All Fields] OR "survive"[All Fields] OR "survived"[All Fields] OR "survives"[All Fields] OR "surviving"[All Fields]) OR "survival rate"[MeSH Terms])) AND (y_10[Filter]).

The Scopus search strategy was: (ALL (oral AND squamous AND cell AND carcinoma OR squamous AND cell AND carcinoma AND of AND head AND neck OR oral AND cancer) AND ALL (surgical AND procedure OR positive AND surgical AND margins OR margins AND of AND excision OR tumor AND free AND margins) OR ALL (negative AND surgical AND margin OR negative AND margins AND of AND excision) AND ALL (neoplasm AND recurrence, AND local OR recurrence OR survival OR survival AND rate)) AND (LIMIT-TO (PUBYEAR , 2025) OR LIMIT-TO (PUBYEAR , 2024) OR LIMIT-TO (PUBYEAR , 2023) OR LIMIT-TO (PUBYEAR , 2022) OR LIMIT-TO (PUBYEAR , 2021) OR LIMIT-TO (PUBYEAR , 2020) OR LIMIT-TO (PUBYEAR , 2019) OR LIMIT-TO (PUBYEAR , 2018) OR LIMIT-TO (PUBYEAR , 2017) OR LIMIT-TO (PUBYEAR , 2016) OR LIMIT-TO (PUBYEAR , 2015))

The Web of Science search strategy was: ALL=(oral squamous cell carcinoma OR squamous cell carcinoma of head and neck OR oral cancer) AND ALL=(Surgical Procedure OR Positive Surgical Margin OR Margins of excision OR Tumor Free Margins) OR ALL=(Negative Surgical Margin OR negative margins of excision) AND ALL=(neoplasm recurrence, local OR survival OR recurrence OR survival rate) AND (2025 OR 2024 OR 2023 OR 2022 OR 2021 OR 2020 OR 2019 OR 2018 OR 2017 OR 2016 OR 2015) AND Web of Science Categories=(Dentistry Oral Surgery Medicine) AND Language=(English).

Additionally, a manual search was conducted on the reference lists of the included articles.

Inclusion and Exclusion Criteria

All studies published within the last 10 years that enrolled patients with OSCC who underwent surgical treatment and reported data on surgical margin status, recurrence rates, and survival outcomes were included.

Studies were excluded if they involved recurrent or non-primary tumors, carcinomas in extraoral locations, patients treated with preoperative radiotherapy and/or chemotherapy. Systematic reviews, case reports, letters to the editor, conference abstracts, expert opinions, books, and studies lacking sufficient data were also excluded.

Study Selection Process

Study selection followed a three-phase process: 1) title screening to exclude duplicates and irrelevant publications, 2) abstract screening based on study design, type of intervention, and outcome variables, and 3) full-text review to confirm eligibility and extract data using a predefined data collection form.

Study selection based on titles and abstracts was conducted independently and in duplicate by two reviewers (AR and AP). Disagreements were resolved by a third reviewer (LB). Inter-rater agreement for inclusion decisions in phases two and three was assessed using Cohen's kappa coefficient.

Data Extraction

Data were extracted and organized in tables according to surgical procedure type (resection with and without safety margins). The following information was collected: Author and year of publication, study design, number of patients, gender, age, tumor location, treatment based on margin status and measurements (positive/negative and in millimeters), recurrence (percentage and/or number), survival rates, and follow-up time in years.

Methodological Quality Assessment:

Risk of bias was independently assessed by two reviewers (AR and AP) to evaluate the methodological quality of the included studies. The Newcastle-Ottawa Scale (17) was used for non-randomized observational studies. Studies scoring &gt;6 `x´ were considered at low risk of bias; those scoring 6 `x´ were considered at high risk.

Inter-rater reliability for quality assessment was measured using Cohen's kappa statistic, following the interpretation scale proposed by Landis and Koch (18).

Certainty of the Evidence

The certainty of evidence (confidence in the effect estimates) was assessed for each outcome using the GRADE (Grading of Recommendations Assessment, Development and Evaluation) approach with GRADEpro GDT software.

Quantitative Synthesis

Odds ratios (OR), Z statistics, p-values, and 95% confidence intervals (CI) were calculated. Forest plots were generated using fixed-effects or random-effects meta-analysis depending on heterogeneity levels. Sensitivity analyses were performed to evaluate the influence of individual studies on the overall effect size.

Heterogeneity and Publication Bias

The statistical heterogeneity of the included studies was assessed using Cochran's Q test and the I² statistic. A p-value &lt; 0.05 was considered statistically significant. I² values of 25%, 50%, and 75% were interpreted as low, moderate, and high heterogeneity, respectively.

The presence of publication bias was evaluated through funnel plot analysis and Egger's regression test.

All statistical analyses were conducted using Comprehensive Meta-Analysis software, version 3.0.

## Results

Study Selection

The literature search strategy identified a total of 878 articles: Medline-PubMed (n=553), SCOPUS (n=62), and Web of Science (n=263). After screening titles and abstracts, 62 studies were selected for full-text review.

**Figure 1:**
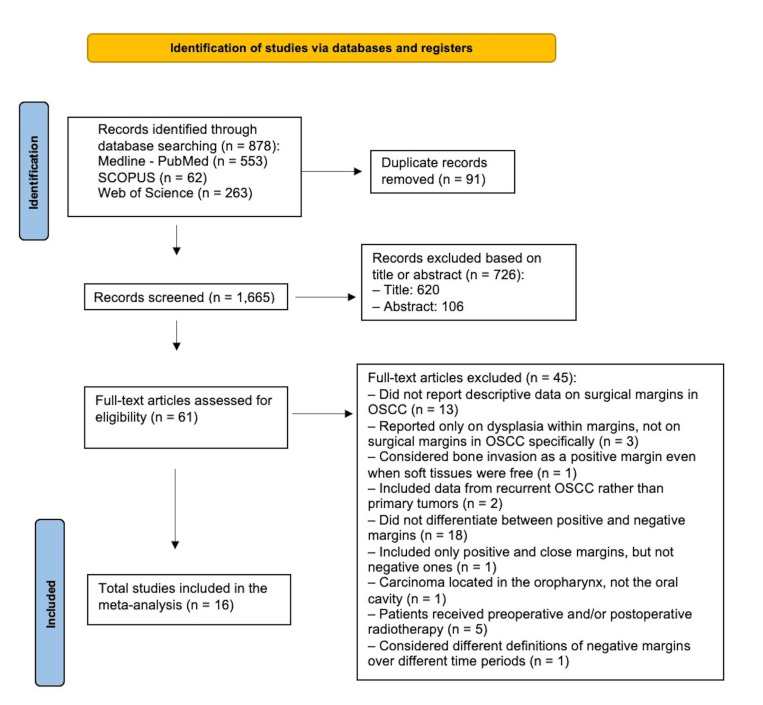
Flow chart detailing exclusion of studies at each stage of screening.

Of these, sixteen studies met the inclusion criteria and were included in the meta-analysis (Figure 1). The level of inter-rater agreement was deemed to be excellent (Cohen's kappa coefficient =0.86).

[caption id="attachment_1875" align="alignnone" width="300"]1 Screenshot[/caption]

Forty-six studies were excluded from the analysis for not meeting the eligibility criteria, mainly due to the lack of quantitative data on surgical margins in OSCC, the selective inclusion of recurrent or extraoral tumors, the absence of differentiation between positive and negative margins, or the inclusion of cohorts treated with preoperative and/or postoperative radiotherapy.

Analysis of Study Characteristics

All included studies reported the status of surgical margins following complete surgical excision of OSCC located at various sites of the oral mucosa. The analysis of patient survival and/or recurrence was based on surgical margin status (positive versus negative) (1 , 2 , 7 , 9 - 11 , 13 , 19 - 27).

Regarding the study's design, fifteen retrospective cohort studies (1 , 7 , 9 - 11 , 13 , 19 - 27) and one prospective cohort study (2) were included.

A total of 20,812 patients were analyzed, with a predominance of males (n=17,234; 82.8%), mostly between the fourth and sixth decades of life, with a mean age of 55.8 years. The tongue was the most frequently affected site of OSCC (Table 1).

Table 1

Most studies have defined a negative margin as a margin greater than 5mm (1 , 7 , 9 - 11 , 13 , 19 - 23 , 25 - 27), except for one study that employed a threshold of &gt;10mm (24). Regarding margin status, 1,442 patients exhibited positive margins, while 10,359 patients exhibited negative margins. Patients with "close" or dysplastic margins were excluded from the statistical analysis (Table 2).

Table 2

Five studies reported the influence of surgical margins on patient survival (1 , 7 , 11 , 20 , 21), and four studies evaluated the impact of margins on local recurrence (9 , 13 , 24 , 25). The remaining studies assessed the effect of margins on both survival and local recurrence of OSCC (2 , 10 , 19 , 22 , 23 , 26 , 27) (Table 2).

Synthesis of Results

Effect of Surgical Margins on Local Recurrence Rate:

A total of 11 studies provided data on the impact of positive versus negative margins on local recurrence rates of OSCC (Table 2). All studies consistently demonstrated that local recurrence rates were significantly higher in patients with positive surgical margins (PM).

Effect of Surgical Margins on Survival Rate

Thirteen studies reported data on the influence of surgical margins on patient survival rates (Table 2). All studies consistently demonstrated that survival rates were significantly higher in patients with negative surgical margins (NM).

Meta-Analysis Results

Odds Ratio of OSCC Recurrence

**Figure 2:**
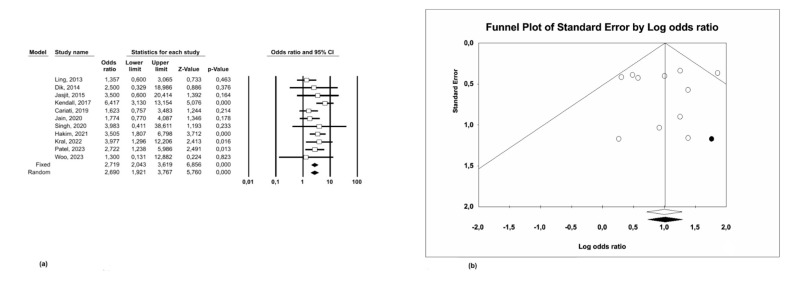
(a) Odds ratio and (b) Funnel Plot for recurrence.

The odds of recurrence were significantly higher in patients with positive margins in comparison to those with negative margins (OR=2.72 95% CI: 2.04 - 3.62 p&lt;0.001) (Figure 2a).

A fixed-effects model was applied due to the low heterogeneity among the included studies (I²=20.95%; p=0.24).

The robustness of the results was supported by sensitivity analysis, in which the meta-analysis outcomes remained consistent when using the "leave-one-out" method. All the results were statistically significant, with none of the confidence intervals including the value of 1 (Figure 4a).

The presence of publication bias was assessed using a funnel plot, which showed a symmetrical distribution around the central axis, suggesting the absence of publication bias (Figure 2b). This result was further confirmed by Egger's regression test (p=0.83).

2

Risk Ratio of Survival

**Figure 3:**
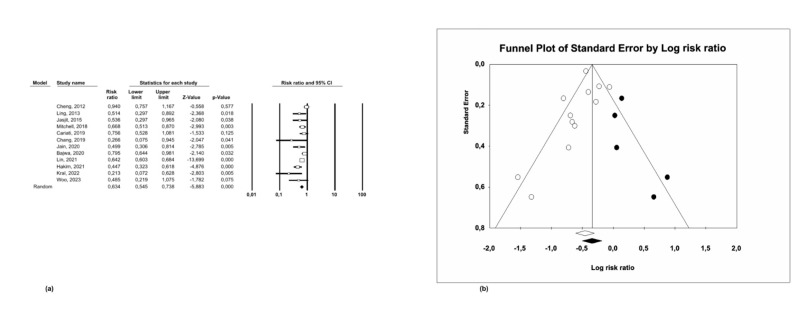
(a) Risk ratio and (b) Funnel Plot of survival.

A risk ratio (RR) of 0.63 was obtained (95% CI: 0.55-0.74; p&lt;0.001), indicating that the presence of negative surgical margins was a protective factor for survival in oral squamous cell carcinoma (Figure 3a).

3

Specifically, patients with negative surgical margins exhibited a 1.58 times greater probability of 5-year survival in comparison to those with positive margins.

A random-effects model was employed due to the moderate heterogeneity that was observed among the included studies (I²=62.54%; p=0.002).

**Figure 4:**
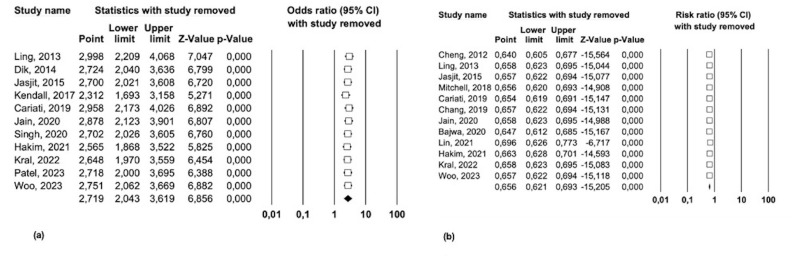
(a) Sensitivity analysis of Odds Ratio and (b) of Risk Ratio for survival.

The findings of the meta-analysis remained consistent following a sensitivity analysis, indicating that the pooled estimates were not driven by any individual study (Figure 4b).

[caption id="attachment_1839" align="alignnone" width="300"]4 Screenshot[/caption]

The presence of publication bias was assessed using a funnel plot, which demonstrated a symmetrical distribution around the central axis, suggesting no evidence of publication bias (Figure 3b).

This result was further substantiated by Egger's regression test, which also indicated the absence of publication bias (p=0.38).

Methodological Quality Assessment

Three studies were considered to have a low risk of bias (7 , 19 , 21), while thirteen were rated as having a moderate risk of bias (1 , 2 , 9 - 11 , 13 , 18 , 20 , 22 , 23 , 25 - 27) (Table 3).

Table 3

The item with the highest risk of bias was "demonstration that the outcome of interest was not present at the start of the study" (Table 3).

The inter-rater agreement for methodological quality assessment, measured using Cohen's kappa (), was 0.9, indicating excellent agreement according to the Landis and Koch scale.

The certainty of the evidence was rated as moderate for both meta-analyses, as shown in Supplementary Table 1 (http://www.medicina.oral.com/carpeta/suppl1_27784).

## Discussion

In this systematic review and meta-analysis, the influence of postoperative surgical margins on local recurrence and overall survival in patients with oral squamous cell carcinoma treated surgically was evaluated.

The integration of sixteen studies involving 20,812 patients demonstrates that positive surgical margins are significantly associated with a higher rate of local recurrence (OR=2.72; 95% CI: 2.04-3.62; p&lt;0.001), while negative margins are a protective factor for overall survival (RR=0.63; 95% CI: 0.55-0.74; p&lt;0.001). These findings remained consistent in sensitivity analyses.

Margins measuring &lt;1mm correlate with higher recurrence rates compared to margins 5mm. This observation lends support to the 5mm safety cutoff that has been proposed in the literature (28 , 29). However, even with margins 5mm, the recurrence risk remains at 20% (4), thereby supporting the indication of adjuvant therapy in certain cases, particularly in cases of aggressive or large tumors (2).

A number of studies have documented recurrence rates of 50% in cases with involved margins, in contrast to the 3.8% observed in cases with clear margins (10 , 13). Loree et al. found rates of 36% versus 18% in positive and negative margins, respectively (32). Zanoni et al. (28) suggested a clear margin of 2.2mm; nevertheless, their study is limited to tongue carcinomas, which restricts its generalizability.

Young K. et al. (33) reported a higher risk of recurrence with margins &lt;5mm (RR: 2.09; 95% CI: 1.53-2.86). However, the findings of Bungum A. et al. (34) and Stathopoulos P. et al. (8) did not reveal significant differences in survival outcomes with margins &lt;5mm. This observation suggests that other prognostic factors, such as nodal involvement or tumor depth, might hold greater influence on patient outcomes compared to margin distance itself.

The location of the tumor has been shown to exert an influence on the probability of recurrence, with tongue cancers exhibiting a more aggressive behavior pattern (24). However, this analysis did not evaluate outcomes based on anatomical subsite. Several studies have confirmed the prognostic value of surgical margins. The 5-year survival rate for patients with negative margins

is 80.8%, while it is a mere 12.5% for those with involved margins (1 , 7). Moreover, positive margins have been associated with an increase in mortality risk up to 2.5 times the baseline value (35).

Binahmed et al. (36) and Chen et al. (31) reported 5-year survival rates of 69% versus 38% and 40% versus 7% for negative and positive margins, respectively. Mitchell et al. (11) observed rates of 81% and 54%. However, the optimal cutoff point for defining a "clear" margin remains controversial. While some authors propose a threshold of 2mm (22), margins &lt;1mm have been associated with reduced survival (21 , 22).

The employment of surgical techniques, such as fragmented resection, may optimize margins without the need for extensive resections (37). Nevertheless, surgery with adequate margins remains the gold standard treatment (1).

Indications for adjuvant therapy are based on histopathological findings, which may include positive margins, perineural or extra nodal invasion, and the depth of invasion (1). McMahon et al. (38) emphasized that regional nodal involvement possesses the potential to be more prognostically significant than margin status.

Advanced age is an adverse prognostic factor, associated with increased postoperative mortality due to functional complications (14). In cases of incomplete resection, adjuvant therapy, either radiotherapy or chemoradiotherapy, has been demonstrated to improve survival outcomes. However, its utilization is often constrained by the occurrence of adverse effects (2 , 12).

The primary methodological limitations of this meta-analysis are the heterogeneity of follow-up periods, the retrospective design of most included studies (15 out of 16), and the limited anatomical representativeness in certain cohorts (22 , 24 , 25). The dichotomous classification employed (positive versus negative margins) was intended to standardize the data, although it may oversimplify a more complex clinical reality.

In order to advance toward a more personalized medicine approach in the management of OSCC, prospective multicenter studies incorporating molecular biomarkers of tumor margins are needed.

## Conclusions

The status of surgical margins has been identified as a predictive prognostic factor in the context of locoregional control and overall survival of patients diagnosed with oral squamous cell carcinoma.

The results of the study indicate that the recurrence rate is higher in positive margins (&lt;5mm) (OR=2.72; 95% CI: 2.04-3.62; p&lt;0.001) and that a negative margin (5mm) is associated with a 1.58-fold greater probability of 5-year survival (RR=0.63; 95% CI: 0.55-0.74; p&lt;0.001). These findings underscore the necessity for oncological resections with adequate margins and support the consideration of adjuvant treatment in cases of close or involved margins.

## Figures and Tables

**Table 1:** Table Characteristics of the patients included in the study.

Study	Country	Number of patients	Age	Gender	Tumor location
Chen et al. (2012)	Taiwan	407 (stages I and II)	>50: 213<50: 194	F: 60 M: 347	Tongue: 208, Buccal mucosa: 137, Floor of mouth: 16, Gingiva: 24, Retromolar trigone: 4, Palate: 11, Lip: 7
Ling et al. (2013)	China	210 (only 179 with margin data)	<45: 59 46-69: 130≥70: 21	F: 97 M: 113	Tongue (total): 210 (lateral borders: 67, base: 69, ventral surface: 28, dorsal surface: 7, other: 39)
Dik et al. (2014)	Netherlands	200	58,9-61,1	F: 87 M: 113	Tongue: 105, Floor of mouth: 73, Buccal mucosa: 22
Dillon et al. (2015)	United States	54	<65: 34 ≥65: 20	F: 14 M: 40	All locations
Tasche et al. (2017)	United States	422	62.14 mean	F: 252 M: 180	Tongue: 190, Gingiva: 89, Floor of mouth: 78, Other: 65
Mitchell et al. (2018)	United Kingdom	591	62 mean (21-96)	F: 203 M: 388	Tongue: 233, Floor of mouth: 130, Other: 228
Cariati et al. (2019)	Spain	200	64,17 mean (19-91)	F: 64 M: 136	Tongue: 87, Floor of mouth: 44, Buccal mucosa: 26, Retromolar area: 18, Other: 25
Chang et al. (2019)	Taiwan	341	52,1 mean (23-84)	F: 28 M: 313	Lip: 2, Retromolar trigone: 16, Gingiva: 43, Tongue: 147, Palate: 7, Buccal mucosa: 115, Floor of mouth: 11
Jain et al. (2020)	India	612	<50: 395 ≥50: 217	F: 184 M: 428	Tongue: 277, Buccal mucosa: 335
Bajwa et al. (2020)	United Kingdom	669 (667 with margin data)	<55: 184 55-64: 208 65-74: 196 ≥75: 79	NS	Tongue: 367, Floor of mouth: 178, Buccal mucosa: 51,Retromolar area: 39, Other: 31
Singh et al. (2020)	India	451	NS	F: 95 M: 334	Tongue: 451
Lin et al. (2021)	Taiwan	15,654	<65: 12,740 ≥65: 2,914	F: 1,503 M: 14,151	Buccal mucosa: 5,240, Lip: 761, Tongue: 5,848, Gingiva: 2,031, Floor of mouth: 481, Palate: 253, Other: 1,040
Hakim et al. (2021)	Germany	753	62 mean (54-72)	F: 269 M: 484	Lip: 21, Tongue: 172, Gingiva: 123, Floor of mouth: 237, Palate: 45, Buccal mucosa: 73, Other: 82
Kral et al.(2022)	Czech Republic	98	60 mean (36-82)	F: 30 M: 68	Tongue: 34, Floor of mouth: 28, Alveolar process: 17, Palate: 8, Retromolar area: 5, Buccal mucosa: 6
Patel et al. (2023)	India	116	43,3 mean	F: 10 M: 106	Buccal mucosa: 116
Woo et al. (2023)	Australia	67	66 mean (32-93)	F: 34 M: 33	Hard palate: 15, Anterior alveolus: 8, Posterior alveolus: 44

NS: Not studied.

**Table 2:** Table Summary of the data extracted in each study.

Study	Number of patients	Negative margins (NM)	Positive margins (PM)	Followup time	Number of local recurrences	Local recurrence rate (%)	Survival rate (%)
Chen et al. (2012)	407 (stages I and II)	>5mm: 362	<1mm: 14	4.3 years mean	NS	NS	5 years>5mm: 91.2%<1mm: 85.1%
Ling et al. (2013)	179	>5mm: 108 (60.3%)	<1mm: 35 (19.6%)	3.05 years mean	>5mm: 30<1mm: 12	5 years:>5mm: 28%<1mm: 34%	5 years>5mm: 55.2%<1mm: 29.2%
Dik et al. (2014)	200	>5mm: 52 (26%)	0mm: 22 (11%)	3 years mean	0mm: 2(9.1%)>5mm: 2(3.8%)	0mm: 9.1%>5mm: 3.8%	NS
Dillon et al. (2015)	54		<1mm: 24 (44%)	2-5 years	5 years <1mm: 121-5mm: 10≥5mm: 2	5 years<1mm: 50% ≥5mm: 22%	5 years<1mm: 42% (10)≥5mm: 78% (7)
Tasche et al. (2017)	422	≥5mm: 110	0mm: 100	5 years	NS	LR Rate (95% CI), 0mm: 44% (34-55) ≥5mm: 11% (6-18)	NS
Mitchell et al. (2018)	591	>5mm: 480	<2mm: 48	NS	NS	NS	5 years>5mm: 81%<2mm: 54%
Cariati et al. (2019)	200	>5mm: 130	<2mm: 34	3 years	>5mm: 46<2mm: 16	>5mm: 35.3%<2mm: 48.3%	>5mm: 66.2% <2mm: 50%
Chang et al. (2019)	341	>5mm: 236	<1mm: 12	4.2 years mean	NS	NS	5 years>5mm: 62.9%<1mm: 12.5%
Jain et al. (2020)	612	>5mm: 496	<1mm: 26	3.35 years mean	>5mm: 114 <1mm: 9	>5mm: 23%<1mm: 35%	>5mm: 77%<1mm: 38%
Bajwa et al. (2020)	667 (stages I and II)	>5mm: 296	<1mm: 68	NS	NS	NS	5 years>5mm: 74%<1mm: 59%
Singh et al. (2020)	451	≥10mm: 1329mm: 378mm: 467mm: 386mm: 965mm: 70	≤1mm: 4	2.6 years mean	≥10mm: 479mm: 148mm: 157mm: 216mm: 475mm: 36≤1mm: 3	NS	NS
Lin et al. (2021)	15,654	≥5mm: 7,243 (46.3%)	0mm: 917 (5.9%)	5 years	NS	NS	≥5mm: 80.8% 0mm: 51.9%
Hakim et al. (2021)	753	≥5mm: 261 (35%)	0mm: 54 (7.2%)	5 years	≥5mm: 35 0 mm: 19	≥5mm: 13% 0 mm: 35%	≥5mm: 23 deaths (8.8%) 0 mm: 22 (41%)
Kral et al. -2022	98	>5mm: 36	0mm: 22	5 years	>5mm: 11 0mm: 14	>5mm: 30.6% 0mm: 63.6%	>5mm: 63.9% 0mm: 13.6%
Patel et al. -2023	116	>5mm: 65	≤5mm: 51	1 year	>5mm: 16 ≤5mm: 24	>5mm: 24.6% ≤5mm: 47%	NS
Woo et al. -2023	67	≥5mm: 56 (83.6%)	<5mm: 11 (16.5%)	4.5 years mean	≥5mm: 4 <5mm: 1	≥5mm: 7.1%<5mm: 9.1%	≥5mm: 75%<5mm: 36%

NS: Not studied.

**Table 3:** Table Risk of Bias Assessment of non-randomized observational studies using the Newcastle-Ottawa Scale-Cohort Studies without control group.

	Representativeness of Cohort	Selection of Non-Exposed Cohort	Ascertainment of Exposure	Demonstration Outcome Not Present at Start	Comparability (Main Factor)	Comparability (Other Factors)	Outcome Assessment	Follow-Up Length Adequate	Adequate Follow-Up Rate	Total score
Chen et al. (2012)	X	X	X	X	X	X	X	X	-	8
Ling et al. (2013)	-	-	X	X	X	X	X	-	-	5
Dik et al. (2014)	-	-	X	X	X	-	X	-	X	5
Dillon et al. (2015)	-	X	-	-	X	X	X	-	X	5
Tasche et al. (2017)	X	-	X	-	X	X	X	X	X	7
Mitchell et al. (2018)	X	-	X	-	X	X	X	X	X	6
Jain et al. (2019)	X	X	-	X	X	X	X	X	X	8
Cariati et al. (2019)	X	-	X	-	X	-	X	X	-	5
Chang et al. (2019)	X	-	X	-	X	X	X	-	X	6
Bajwa et al. (2020)	X	X	X	-	X	X	X	-	-	6
Singh et al. (2020)	-	X	X	-	X	-	X	X	X	6
Lin et al. (2021)	X	X	X	-	X	X	X	X	X	8
Hakim et al. (2021)	X	X	X	-	X	X	X	X	-	7
Kral et al. (2022)	-	-	X	-	X	X	X	X	X	6
Patel et al. (2023)	-	-	X	-	X	X	X	-	X	5
Woo et al. (2023)	-	-	X	-	X	X	X	X	X	6

3

## Data Availability

Declared none.
